# The risks of exclusive reliance on rapid diagnostic tests for malaria in Angola

**DOI:** 10.11604/pamj.2025.52.149.50378

**Published:** 2025-12-09

**Authors:** Hermenegildo Osvaldo Chitumba

**Affiliations:** 1 Department of Pathology and Diagnostic Methods, School of Medicine, José Eduardo dos Santos University, Huambo, Angola,; 2 Global Health and Tropical Medicine, "Instituto de Higiene e Medicina Tropical, Universidade Nova de Lisboa", Lisboa, Portugal

**Keywords:** Malaria, *Plasmodium*, Angola

## Abstract

Malaria remains a major burden in Angola, where reliance on rapid diagnostic tests limits species identification and parasite quantification. Strengthening microscopy and integrating molecular diagnostics are essential to improve clinical management, surveillance capacity, and detection of emerging resistance.

## Commentary

According to the 2024 World Malaria Report [1], the World Health Organization (WHO) African region remains the most affected by the disease, accounting for approximately 94% of malaria cases and 95% of the estimated malaria deaths worldwide in 2023. Angola continues to face one of the highest malaria burdens in sub-Saharan Africa, with the disease remaining a major public health concern. In 2024, over 7 million malaria cases and approximately 11,000 deaths were reported, primarily affecting children and pregnant women [2].

However, malaria diagnosis in most health facilities remains almost entirely reliant on rapid diagnostic tests (RDTs) [3]. According to Rowe *et al*. [4], a survey of 33 outpatient facilities found that only around 39% conducted both microscopy and RDTs concurrently. RDTs are widely used due to their ease of use and rapid turnaround; however, they are limited in that they do not allow parasite quantification or precise species identification, thereby constraining certain aspects of clinical management [5,6].

Although RDTs have transformed case management in resource-limited settings, their exclusive use raises significant clinical and public health concerns that require urgent attention. Their predominance has contributed to the gradual abandonment of microscopy in peripheral and even provincial hospitals [7]. Microscopy remains the gold standard for parasite quantification, species identification, and detection of non-*falciparum* infections [5], as well as for monitoring treatment efficacy and identifying atypical presentations. The lack of microscopy and/or molecular diagnostic techniques for malaria reduces the health system´s capacity to detect treatment failures or emerging resistance patterns, increases the risk of both overdiagnosis and underdiagnosis, contributes to the mismanagement of bacterial, viral, or zoonotic infections presenting with similar symptoms, and weakens the national epidemiological surveillance system.

A review of the scientific literature and epidemiological surveillance reports reveals that Angola continues to experience substantial gaps in epidemiological data, with a scarcity of comprehensive information on parasite density trends, the prevalence of mixed infections (co-infections with different *Plasmodium* species), and regional variation in *Plasmodium* species distribution.

We advocate for the implementation of a balanced malaria diagnostic strategy that maintains the central role of microscopy while integrating targeted interventions, including the reintroduction and strengthening of microscopy services in municipal and provincial hospitals, the establishment of quality control mechanisms for RDT use, and the training of laboratory personnel in both malaria microscopy and differential diagnosis. Concurrently, investment in molecular diagnostics should be prioritized to enable accurate monitoring of *Plasmodium* species, parasite density, and submicroscopic infections.

## Available online at

https://www.panafrican-med-journal.com//content/article/52/149/full

## References

[1] World Health Organization (2025). World malaria report 2024.

[2] World Health Organization (2025). Angola Observes World Malaria Day with Focus on Renewed Commitment.

[3] Lopes SC, Mugizi R, Pires JE, David F, Martins J, Dimbu PR (2020). Malaria Test, Treat and Track policy implementation in Angola: a retrospective study to assess the progress achieved after 4 years of programme implementation. Malar J.

[4] Rowe AK, de León GF, Mihigo J, Santelli AC, Miller NP, Van-Dúnem P (2009). Quality of malaria case management at outpatient health facilities in Angola. Malar J.

[5] Mediavilla A, Silgado A, Febrer-Sendra B, Crego-Vicente B, Martínez-Vallejo P, Maturana CR (2024). Real-time PCR for malaria diagnosis and identification of Plasmodium species in febrile patients in Cubal, Angola. Parasit Vectors.

[6] Febrer-Sendra B, Crego-Vicente B, Nindia A, Martínez-Campreciós J, Aixut S, Mediavilla A (2023). First field and laboratory evaluation of LAMP assay for malaria diagnosis in Cubal, Angola. Parasit Vectors.

[7] Kerr G, Wini L, Leaburi J, Macdonald J, Russell TL (2025). Utility of rapid diagnostic tests and microscopy to detect malaria in health facilities across the Solomon Islands. Malar J.

